# A Tale of Two Islands – Leptospirosis in Strikingly Different Ecological Settings Reveals Pathogenomic Insights

**DOI:** 10.4269/ajtmh.24-0254

**Published:** 2024-05-02

**Authors:** David A. Haake

**Affiliations:** VA Greater Los Angeles Healthcare System and the David Geffen School of Medicine at UCLA, Los Angeles, California

Leptospirosis is a widespread zoonosis caused by bacteria belonging to an ancient group of highly motile spirochetes. More than one million cases and 60,000 deaths from leptosporosis are estimated to occur annually.^1^ Many wild and domestic animals serve as hosts that pass infection to humans via direct or indirect exposure to abraded skin or mucous membranes. After penetrating host tissues, pathogenic leptospires evade host defenses and achieve remarkably high bloodstream levels (as high as 10^6^ cfu/mL).^2^^,^^3^ Rapid dissemination to multiple organs, including kidney, liver, lung, and brain, occurs, indicating the ability to migrate across the bloodstream barrier with ease. The resulting pro-inflammatory host response can result in organ failure, hemorrhagic pneumonia, and occasionally, death.

Little is known about why some persons develop relatively mild, self-limited infection while others have severe, life-threatening outcomes due to leptospirosis. It is likely that both infection risk and severity depend on a combination of factors including host susceptibility, the epidemiologic setting in which exposure occurs, and the virulence of the infecting strain.^4^ Host susceptibility is likely affected by skin integrity, duration of exposure, protective clothing and footwear, genetic factors, comorbidities, and age. While younger adults are at higher risk of infection for vocational reasons, older adults are at higher risk of mortality.^5^ Epidemiologic factors that affect the level of exposure, such as density and type of reservoir hosts, occupation, housing, rainfall, and flooding, are well known to be important determinants of risk of infection and may also impact severity. The epidemiologic setting also determines what leptospiral species and strains are present when exposure occurs, with *Leptospirosis interrogans* the predominant species associated with severe outcomes.

Leptospirosis risk factors and their impact on patient outcomes are dramatically illustrated in the study by Desmoulin et al.^6^ published in this month’s issue of the *American Journal of Tropical Medicine and Hygiene.* The study describes the clinical features of 490 leptospirosis cases on Mayotte and Reunion, two islands in the Indian Ocean. Overall, 20% of cases were defined as severe based on death of the patient or the requirement for vasopressor support, mechanical ventilation, renal dialysis, or transfusion. Fortunately, only 1% of cases were fatal, likely due to the high level of awareness regarding leptospirosis on the islands, resulting in early initiation of antibiotic therapy and other management interventions. In 142 samples, the authors were able to amplify leptospiral DNA and sequence six leptospiral genes for multilocus sequencing typing to identify the infecting species.

Leptospirosis severity and the causative species on the islands of Reunion and Mayotte were strikingly different. Leptospirosis on Reunion had a relatively high 28% severity rate, and 80% (81 of 102) of infections were caused by *L. interrogans*. In contrast, only 10% of leptospirosis on Mayotte was severe, and 90% (36 of 40) of infections were caused by species other than *L. interrogans*. Hospitalization and liver, lung, and kidney function impairment were each more common in patients on Reunion than on Mayotte. While the authors identified a number of additional risk factors including age, tobacco use, and alcohol use that could account for the differences in outcomes between the two islands, this study offers some of the best evidence that the causative leptospiral species is a major contributor to leptospirosis severity.

Recently, there has been an explosion of knowledge about the number and complexity of leptospiral species and strains that exist in nature. Almost 70 species have now been isolated and reported, reflecting the adaptation of leptospires to a diverse array of environmental and host niches. The number of reported species is certain to increase given the availability of selective media that enhance recoverability. Leptospiral species identified thus far reveal a broad range of organisms that fall into four clades: Clades S1 and S2 contain environmentally adapted saprophytic species, while clades P1 and P2 contain species with the potential to cause infection in animals.^7^ The most virulent organisms are found within the P1+ subgroup of clade P1, and most human infections are caused by the archetypal P1+ subgroup member *L. interrogans*. However, other members of the P1 and P2 clades have been associated with a significant number of human cases.

Comparative analysis of leptospiral genomes has begun to reveal details of the evolutionary path from a free-living ancestor to the most virulent species, *L. interrogans*.^7^^–^^12^ Along the way, genes involved in microbial metabolism have been exchanged for those enabling host adaptation. Ready acquisition of new genes is reflected in the fact that leptospires have an open pan-genome, meaning that so many unique genes are found in each new sequenced genome that predicting the total number of leptospiral genes across the genus is impossible. Interestingly, the P1 species have the highest amount of genomic “openness” (genes unique to each species), suggesting that the ability to acquire new genes allowed these organisms to adapt to animal hosts.^7^

Identification of the key genes accounting for leptospiral virulence is of great interest. Genomic comparisons, combined with phenotypic studies, have begun to yield answers to the question of what genes are associated with infection. Some virulence genes that have been identified encode proteins required for defense against oxidative stress,^13^ resistance to complement-mediated killing,^14^ and toxin production.^15^ However, a large number of the genes associated with infectivity and virulence encode proteins of unknown function. One way to try to identify genes that are of greatest interest is by comparing the leptospiral transcriptome outside vs inside the host; genes that are strongly upregulated during host infection are of greatest interest. Another approach is to knock out or knock down candidate virulence genes and assess the virulence of the resulting organism, though this approach may fail to identify virulence genes from pathogenic leptospires with redundant virulence mechanisms.

Given that *L. interrogans* and other P1+ subgroup members contain a large number of genes not found in other leptospires, how can we determine which of these genes encode proteins responsible for their increased virulence? Highly relevant insights are provided by clinical studies such as that by Desmoulin et al.^6^ that compare patient outcomes for infections caused by different leptospiral species. Clinical samples from such studies can even be used to detect, amplify, and sequence candidate virulence genes. DNA capture and enrichment techniques have been developed for characterizing the genomic content of leptospiral species in clinical samples, even when leptospiral nucleic acids are present below the threshold for PCR and leptospiral cultivation is unsuccessful.^16^ Such approaches greatly expand our ability to study the molecular epidemiology of leptospirosis across broad geographical areas and multiple host species.^17^ Deciphering pathogenomic determinants is especially powerful when applied to well-characterized samples containing diverse leptospiral strains from patients with different levels of clinical severity. For as Maya Angelou said, “In diversity there is beauty and there is strength.”
